# Ferroptosis in pulmonary fibrosis: an emerging therapeutic target

**DOI:** 10.3389/fphys.2023.1205771

**Published:** 2023-08-17

**Authors:** Chunyan Wang, Shucheng Hua, Lei Song

**Affiliations:** ^1^ Department of General Practice, Key Laboratory of Organ Regeneration and Transplantation of the Ministry of Education, The First Hospital of Jilin University, Changchun, China; ^2^ Department of Respiratory Medicine, Center for Pathogen Biology and Infectious Diseases, The First Hospital of Jilin University, Changchun, China

**Keywords:** GPx4, natural compounds, p53, lipid peroxides, Nrf2

## Abstract

In recent years, the role of ferroptosis in pulmonary fibrosis has garnered increasing interest as a potential therapeutic target. Pulmonary fibrosis is a pathological process characterized by the accumulation of extracellular matrix in affected lung tissues, and currently, there are no effective therapies for preventing or reversing the fibrotic lesions. Ferroptosis is a form of programmed cell death that is regulated by a network of enzymes and signaling pathways. Dysregulation of ferroptosis has been implicated in several diseases, including pulmonary fibrosis. The accumulation of lipid peroxides in the course of ferroptosis causes damage to cell membranes and other cellular components, leading ultimately to cell death. Relevant targets for therapeutic intervention in ferroptosis include key enzymes, such as glutathione peroxidase 4, transcription factors like nuclear factor erythroid 2-related factor 2, and iron chelation. This review provides an overview of the emerging role of ferroptosis in pulmonary fibrosis and highlights potential therapeutic targets in this pathway. Further research is needed to develop safe and effective approaches targeting ferroptosis in treatment of pulmonary fibrosis.

## 1 Introduction

Pulmonary fibrosis (PF) is a chronic and progressive lung disease that involves the scarring of lung tissue, leading to the impairment of lung function (Wijsenbeek et al., 2022). The term “fibrosis” refers to the process of tissue damage, inflammation, and scarring, which is caused by the excessive deposition of extracellular matrix proteins like collagen. There are many different forms of PF, including idiopathic pulmonary fibrosis (IPF), which is the most common type and is characterized by the presence of fibrotic changes in the lung tissue without any known cause (Wijsenbeek et al., 2022).

Therapy for pulmonary fibrosis involves a combination of medical interventions, including pharmacological therapies, oxygen therapy, and pulmonary rehabilitation (Liu G. Y. et al., 2022). There is no cure for PF, and the available treatments can only slow the progression of the disease. Pharmacological therapies include immune suppressants, such as prednisone, immunomodulatory agents, such as mycophenolate mofetil, and antifibrotic agents, such as pirfenidone and nintedanib (Liu G. Y. et al., 2022). Despite the availability of several treatment options, there remain several challenges and dilemmas in the management of pulmonary fibrosis. One major dilemma is the limited efficacy of available treatments. Although pharmacological therapies and supplemental oxygen therapy can slow the progression of the disease, they do not offer a cure or complete reversal of the damage. In addition, these treatments are often accompanied by significant side effects, which can reduce their overall effectiveness or limit their use.

Finally, there is a need for more research to understand the underlying pathogenesis of PF and to identify new therapeutic targets. This includes research into the role of epigenetics, proteomics, and immunology in the development and progression of PF (Wijsenbeek et al., 2022). In recent years, researchers have begun to explore the potential of novel pathways, such as ferroptosis, as emerging therapeutic targets in fibrosis (Huang et al., 2023). A better understanding of the pathogenesis of PF and the development of novel therapies could help to alleviate the significant burden of fibrotic diseases on patients and healthcare systems worldwide.

## 2 Overview of ferroptosis and its signal transduction

Ferroptosis is a novel form of cell death that has been identified in recent years. This type of cell death is characterized by the buildup of iron and lipid peroxides within the cell (Jiang et al., 2021). Ferroptosis can occur in response to various stimuli such as exposure to toxins, starvation, and oxidative stress, leading to the accumulation of iron in the cell (Jiang et al., 2021). During ferroptosis, the iron can react with hydrogen peroxide and lipid peroxides to form reactive oxygen species or reactive oxygen species-like that can build up inside the cell, leading to lipid peroxidation, and ultimately, the breakdown of crucial cellular membranes and cell death (Jiang et al., 2021).

**FIGURE 1:**
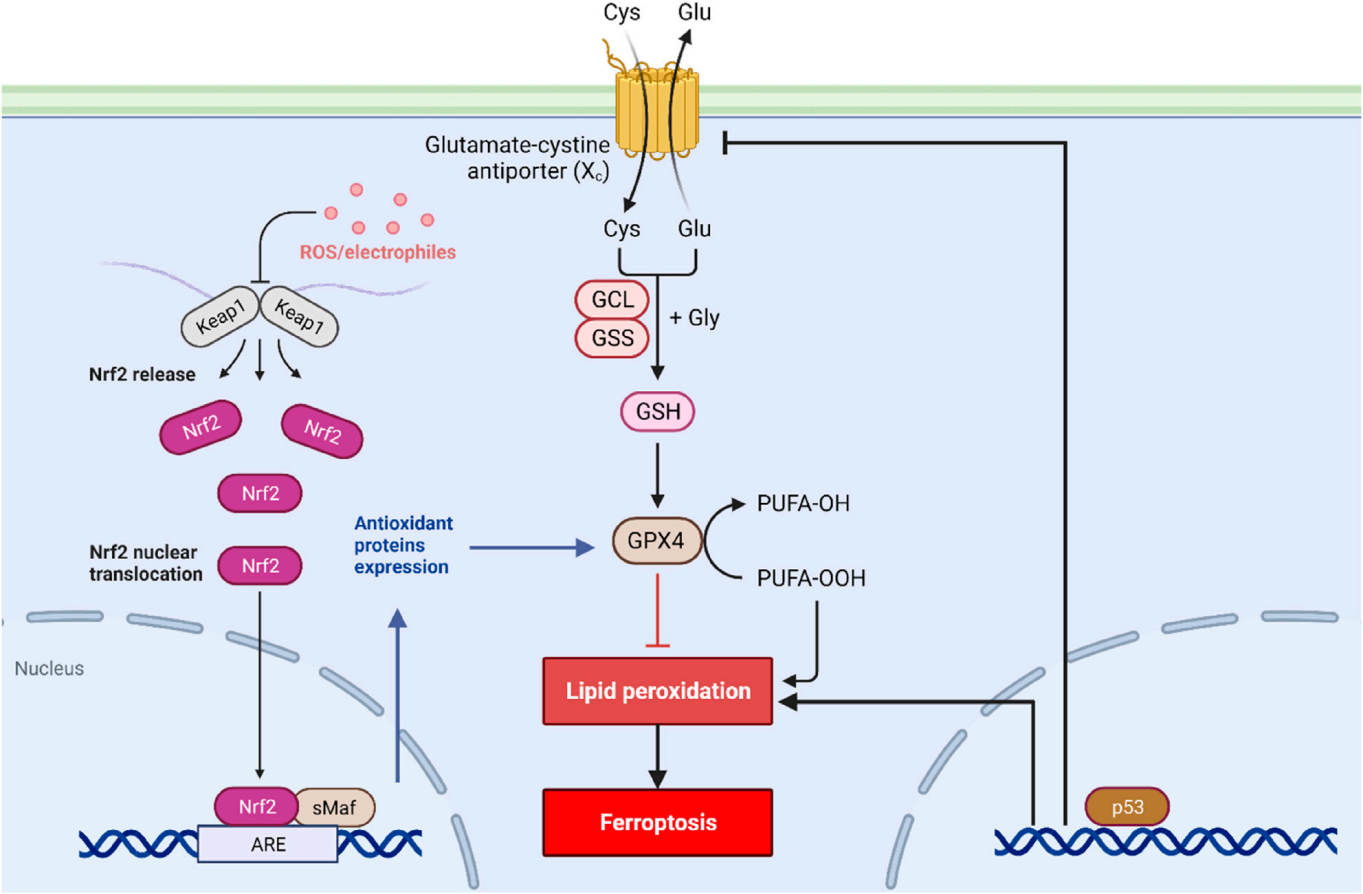
Signal transduction of ferroptosis. Ferroptosis is initiated by the depletion of GSH and iron accumulation in the cytoplasm. Along with this, the activity of the cystine/glutamate antiporter system is blocked, leading to decreased GSH production and increased oxidative stress. The P53 and Nrf2 pathways also play a critical role in regulating ferroptosis.

Numerous researchers have described the signaling cascades involved in ferroptosis (Figure 1), emphasizing various NADPH-dependent enzymes, such as G6PD, glutathione peroxidases, and phospholipid hydroperoxide glutathione peroxidase (GPx4) (Jiang et al., 2021). Furthermore, numerous molecular targets have been identified that can influence iron metabolism, lipid peroxidation, and reactive oxygen species biochemistry, and anti-oxidative defense mechanisms. These kinds of factors include various transcription factors such as Nuclear Factor-erythroid 2 like-2 (Nrf2), P53, and the Protein Kinase R-like endoplasmic reticulum kinase (PERK) (Hu et al., 2022). Nrf2 is a vital transcription factor in the cellular defense against oxidative stress, and its regulation is one of the main mechanisms to prevent ferroptosis (Hu et al., 2022). Studies have shown that during the process of ferroptosis, Nrf2 levels are decreased, leading to greater oxidative stress and ultimately the promotion of cell death (Hu et al., 2022). Kelch-like ECH-associated protein 1 (KEAP1) is also critical in the process of regulating ferroptosis, given that KEAP1 regulates Nrf2 protein stability and turnover by its interaction with Cullin 3 (Koppula et al., 2022). The role of P53 is also an important one in the context of ferroptosis since it can regulate cell survival or death programs, including mechanisms such as apoptosis, autophagy, and senescence (Liu and Gu, 2022). Recent studies have highlighted that the loss of P53 can increase cellular susceptibility to ferroptosis, which can also contribute to the development of various cancers (Liu and Gu, 2022).

## 3 Ferroptosis and PF

In the context of pulmonary fibrosis, accumulating evidence supports the concept that ferroptosis plays a critical role in driving the fibrotic process (He et al., 2021; Li et al., 2021). Studies have shown increased levels of iron and iron-related proteins in fibrotic lungs, indicating disrupted iron homeostasis (Neves et al., 2017; Cheng et al., 2021; Yue et al., 2023). Iron deposition can trigger the generation of ROS, leading to oxidative stress and lipid peroxidation. The accumulation of lipid peroxides is directly correlated with the severity of fibrotic lesions and lung function decline. Moreover, the excessive lipid peroxidation products exacerbate inflammation, initiate fibrogenesis, and contribute to tissue damage, perpetuating the vicious cycle of fibrosis (Cheng et al., 2021). Studies have shown that the accumulation of iron within the lung tissues may be influenced by two primary mechanisms: an increased rate of heme degradation within the pulmonary macrophages (Neves et al., 2019), leading to increased iron levels, and an increase in the expression of divalent metal transporter 1 (DMT1), which facilitates the uptake of iron into lung cells (Ghio et al., 2013; Ali et al., 2020).

Several molecular mechanisms have been implicated in the relationship between ferroptosis and pulmonary fibrosis. One key player is the antioxidant enzyme glutathione peroxidase 4 (GPX4), which plays a crucial role in inhibiting lipid peroxidation and suppressing ferroptotic cell death (Liu M. et al., 2022; Rochette et al., 2022). GPX4 utilizes reduced glutathione as a reducing agent to remove lipid hydroperoxides from cellular membranes (Forcina and Dixon, 2019; Li et al., 2022). Reduced expression or activity of GPX4 has been observed in fibrotic lung tissue, reducing its ability to detoxify lipid peroxides and rendering cells more vulnerable to ferroptosis (Tsubouchi et al., 2019). The loss of GPX4 function is associated with increased lipid peroxidation and the progression of fibrosis (Tsubouchi et al., 2019). Other regulators of ferroptosis have also been studied in the context of pulmonary fibrosis. One example is the transcription factor Nrf2, which has been shown to play a critical role in cellular defense against oxidative stress. Studies have shown that Nrf2 activity is decreased in patients with pulmonary fibrosis (Chen et al., 2021), indicating that the loss of Nrf2-mediated defense could encourage ferroptosis in pulmonary tissue. Research has also shown that pharmacological manipulation of the cystine transporter Slc7a11/xCT reversed and ameliorated the profibrotic and senescence phenotype of old lung fibroblasts (Ritzenthaler et al., 2022).

Recent studies have examined the possible utility of ferroptosis-targeting therapies as potential treatments for pulmonary fibrosis. One of the leading causes of pulmonary fibrosis is Idiopathic Pulmonary Fibrosis (IPF) that lacks a precise therapeutic intervention. A study conducted in bleomycin-induced pulmonary fibrosis mice, showed marked deposition of iron and elevated levels of ferroptosis markers (Cheng et al., 2021). Another study reported that the methylation regulator Uhrf1 (Ubiquitin Like with PHD and Ring Finger Domains 1), upregulated in mouse models of pulmonary fibrosis, that promotes lung ferroptosis via epigenetic repression of GPX4 and FSP1 genes (Liu Y. et al., 2022). According to these observations, Ferroptosis inhibition could be a potential therapy for IPF. Several approaches have been explored to reduce ferroptosis-induced pulmonary fibrosis, including antifibrotic treatment and the use of inhibitors of ferroptosis-inducing enzymes (Pei et al., 2022). An example of this approach is the use of Liproxstatin-1, a potent inhibitor of ferroptosis that has been shown to decrease lung fibrosis in bleomycin-induced IPF mice, suggesting that targeting ferroptosis may be a novel approach to treating pulmonary fibrosis (Pei et al., 2022).

## 4 Natural compounds targeting ferroptosis in treatment of fibrosis

Due to the pivotal role of ferroptosis in the pathogenesis of fibrosis, natural compounds that target ferroptosis could be promising therapeutic agents for the treatment of fibrosis. Recent studies have identified several natural compounds with ferroptosis inhibitory properties (Soriano-Castell et al., 2021). These compounds target various molecular pathways involved in ferroptosis, including lipid peroxidation, iron metabolism, glutathione utilization, and antioxidant defense (El Hajj et al., 2023). Among these compounds, polyphenols, flavonoids, and terpenoids have emerged as potential therapeutic agents for the treatment of fibrosis (Wu et al., 2022; Yang et al., 2022; El-Horany et al., 2023; Wang et al., 2023; Zhu et al., 2023). One example of a natural compound with ferroptosis inhibitory properties is Taxifolin, a polyphenol found in milk thistle seeds, Chinese yew, and several other plants. Taxifolin has been shown to attenuate fibrosis in animal models of silica-induced lung fibrosis through inhibition of lipid peroxidation and regulation of iron metabolism (Yuan et al., 2022). Similarly, *Tripterygium wilfordii* Hook. f., has been shown to reduce Paraquat induced acute lung injury and fibrosis, by inhibiting ferroptosis through the modulation of iron metabolism and the upregulation of antioxidant defense pathways (Song et al., 2023). Another natural compound with ferroptosis inhibitory properties is Virofree, an Herbal Medicine-Based Formula. Virofree has been shown to inhibit ferroptosis that occurs in SARS-CoV-2 patients via attenuating cellular iron accumulation (Doan et al., 2022). Additionally, Virofree was also able to reduce the expression levels of fibrosis-related genes *in vitro* (Doan et al., 2022).

Despite the promising preclinical data, the clinical benefits of natural compounds targeting ferroptosis in fibrosis remain to be fully elucidated. There is a need for more extensive preclinical studies and clinical trials to establish optimal doses, efficacy, and safety of these natural compounds. Moreover, the use of natural compounds as ferroptosis inhibitors may require a combination with established therapies or novel genetic approaches to enhance efficacy in the treatment of fibrosis.

## 5 Conclusion and future perspective

In conclusion, ferroptosis is emerging as a promising therapeutic target for the treatment of fibrosis. This process contributes to the development of fibrosis by promoting the death of fibroblasts and the activation of inflammatory responses. Several natural compounds and pharmacological agents have been identified as potential ferroptosis inhibitors. These compounds target various molecular pathways involved in ferroptosis, including lipid peroxidation, iron metabolism, and antioxidant defense. Preclinical evidence suggests that these agents have promising therapeutic potential in the treatment of fibrosis, although there is still limited knowledge on their long-term safety and efficacy.

Future perspectives for the targeting of ferroptosis in fibrosis include the development of more specific ferroptosis inhibitors, the identification of novel molecular targets, and the development of more precise and personalized treatment approaches. Clinical trials are needed to evaluate the safety and efficacy of targeted ferroptosis inhibitors in humans and to identify optimal dosages and delivery methods. Moreover, there is a need for the identification of biomarkers to monitor disease progression and treatment response, especially in the case of idiopathic or non-specific fibrosis. With the increasing understanding of the role of ferroptosis in fibrosis and the availability of novel pharmacological tools, the potential for targeted therapies for fibrosis is optimistic. Therefore, further studies are encouraged to uncover the potential of ferroptosis modulation as an effective therapeutic approach in the management of fibrosis.
